# Inflammatory Cloacogenic Polyp: A Rare Benign Colorectal Polyp

**DOI:** 10.7759/cureus.22014

**Published:** 2022-02-08

**Authors:** Neha Prakash, Shiv J Vyas, Abdul Mohammed, Nisheet Waghray

**Affiliations:** 1 Medicine, A.T. Still University, School of Osteopathic Medicine, Mesa, USA; 2 Medicine, California Northstate University, Elk Grove, USA; 3 Hospital Medicine, Cleveland Clinic, Cleveland, USA; 4 Gastroenterology and Hepatology, Case Western Reserve University School of Medicine/Metrohealth Medical Center, Cleveland, USA

**Keywords:** solitary rectal ulcer, mucosal prolapse, colorectal polyp, benign tumors, colonoscopy

## Abstract

Inflammatory cloacogenic polyps are a rare kind of benign polyp that is located in the anal transitional zone and rectum. We report the case of a 53-year-old male who underwent a diagnostic colonoscopy for a positive fecal immunochemical test. Two 7 mm polyps were found in the rectum with a pathological diagnosis of inflammatory cloacogenic polyp. The polyp was endoscopically resected. These polyps are associated with chronic inflammatory conditions such as Crohn’s disease and colorectal tumors. Because of malignant transformation potential, inflammatory cloacogenic polyps are endoscopically removed.

## Introduction

Inflammatory cloacogenic polyps are a rare type of anorectal polyp that usually arise in the anal transitional zone [1]. They share distinctive morphologic and histological characteristics with other disorders included in the mucosal prolapse syndromes. Given the paucity of literature regarding inflammatory cloacogenic polyps, the etiopathogenesis of these lesions is poorly defined. While a majority of patients have symptoms, 20% of cases remain asymptomatic [2]. We present a case of inflammatory cloacogenic polyp in an asymptomatic patient with a positive fecal immunochemical test (FIT) performed for colorectal cancer screening.

## Case presentation

History

A 53-year-old female with a history of low-grade papillary tumor of the bladder, cigarette smoking, and alcohol abuse was seen in the gastroenterology clinic for colorectal cancer screening. She denied any current symptoms of abdominal pain, nausea, vomiting, hematemesis, melena, hematochezia, constipation, diarrhea, tenesmus, fecal urgency, fevers, chills, loss of appetite, or weight loss. She had no family history of gastrointestinal malignancy or inflammatory bowel disease.

Physical examination

On physical exam, we did not elicit any abdominal tenderness. There was no organomegaly or abdominal distention. Normal bowel sounds were heard all over the abdomen. A direct rectal exam was positive for dark-colored stool on the gloved finger.

Investigations

**Figure 1:**
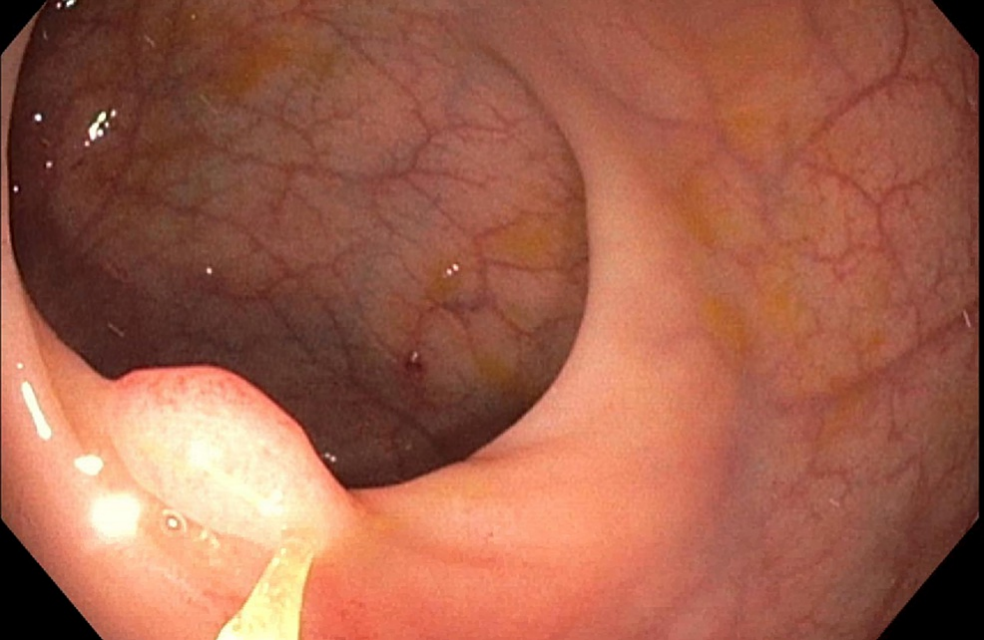
Endoscopic appearance of inflammatory cloacogenic polyp on diagnostic colonoscopy

**Figure 2:**
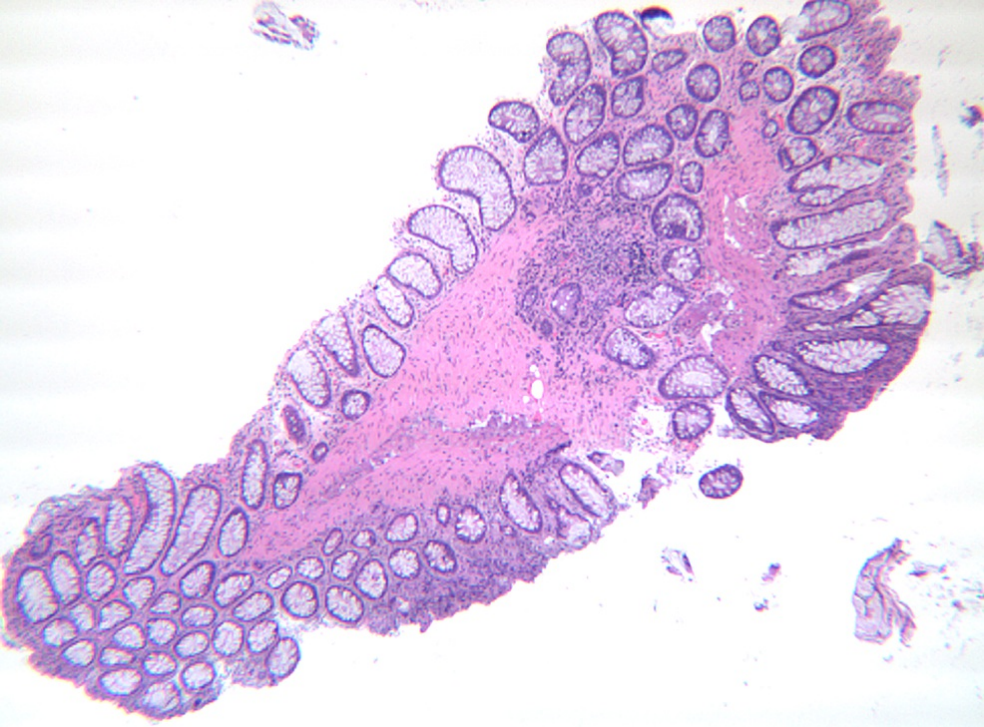
Histological appearance of cloacogenic polyps with an inflamed fibromuscular stroma and hyperplastic epithelium

Laboratory studies revealed a hemoglobin 12.2 g/dl, hematocrit 40%, MCV 88.3 fL, platelet 368 k/uL. She tested positive on a FIT and subsequently underwent a diagnostic colonoscopy two weeks later. The colonoscopy revealed two sessile 7 mm polyps in the rectum (Figure 1). The sigmoid colon had a total of seven polyps ranging in size from 15 mm to 25 mm with all of the polyps removed using snare polypectomy. Histopathology findings demonstrated prolapse-induced inflammatory cloacogenic polyp in the rectum (Figure 2). Histopathology of the polyps in the sigmoid colon was consistent with tubular adenoma and serrated adenoma.

Treatment plan

After polyp resection, the patient continues to be asymptomatic. Since cloacogenic polyps are benign, we did not pursue any further management. However, due to the presence of a tubular adenoma, the patient is scheduled to follow up in the gastroenterology clinic for endoscopic surveillance.

## Discussion

Inflammatory cloacogenic polyps were first described by Lobert et al. in 1981 [1]. They are single or multiple, more pedunculated than sessile, 10 mm to 50 mm in size, located at the anorectal junction [3]. An inflammatory cloacogenic polyp may morphologically mimic hemorrhoid, solitary rectal ulcer, villous adenoma, or anorectal carcinoma. Hence, a histopathological evaluation is essential to distinguish it from carcinoma and squamous metaplasia of chronically prolapsing hemorrhoids [4]. Histological findings are a tubulovillous growth pattern and irregular-shaped crypts displaced into the submucosa, surrounded by a prominent fibromuscular stroma with superficial ulceration. There is a distinct mixed inflammatory infiltrate of the fibromuscular stroma. The cysts are lined by colonic epithelium, without dysplasia [1]. The diagnosis of inflammatory cloacogenic polyp is confirmed by the presence of squamous or transitional epithelium overlying the polyp. Inflammatory cloacogenic polyp shares the hallmark histological feature of crypts displaced into the submucosa with the solitary rectal syndrome. However, unlike inflammatory cloacogenic polyps that are found in the anal transitional zone, solitary rectal ulcers occur in the rectum.

Cloacogenic polyps are relatively more common in women between 40 and 60 years of age [4]. Hematochezia is the most common clinical presentation of cloacogenic polyps. In addition to rectal bleeding, other symptoms associated with inflammatory cloacogenic polyps are constipation, tenesmus, excessive straining, anal swelling, and anal itching. However, 20% of patients can also be asymptomatic [2].

The pathophysiology of cloacogenic polyps remains unclear. However, since it shares histological features with other causes of mucosal prolapse syndromes, such as solitary rectal ulcer syndrome, repeated mucosal ischemia and regeneration are postulated to play a role in pathogenesis [2,5]. Solitary rectal ulcer syndrome is associated with excessive straining on defecation, the inability of the pelvic floor musculature to relax, leading to mucosal prolapse and ischemia [6]. Although half of the inflammatory cloacogenic polyps are associated with mucosal prolapse, the effect of stercoral forces in the formation of cloacogenic polyps is unknown.

Inflammatory cloacogenic polyps have been reported in adults with various gastrointestinal disorders, including Crohn’s disease, malabsorptive states, diverticulosis, colorectal tumors, and hemorrhoids. However, a causal relationship between the diseases and cloacogenic polyps has not been identified. Inflammatory cloacogenic polyps have also been associated with dysplastic lesions in the anus and anal intraepithelial neoplasia [5,7]. Human papillomavirus, routinely implicated in anorectal neoplasms, is also related to inflammatory cloacogenic polyps [7].

Endoscopic or surgical resection of lesions in addition to a high fiber diet is the preferred treatment [3,4]. Routine endoscopic surveillance is highly encouraged after polypectomy because of the increased risk of recurrence and malignant transformation [7].

## Conclusions

In conclusion, inflammatory cloacogenic polyps are rare, benign, polypoidal lesions that can be found on colonoscopy. Since they can be mistaken for anorectal neoplasm or hemorrhoids, endoscopists need to be familiar with inflammatory cloacogenic polyps. Active surveillance after resection is necessary because of their high risk of recurrence and potential for malignant transformation. 
